# Comparison between electromagnetic transponders and radiographic imaging for prostate localization: A pelvic phantom study with rotations and translations

**DOI:** 10.1002/acm2.12119

**Published:** 2017-07-12

**Authors:** Daniel G. Hamilton, Dean P. McKenzie, Anne E. Perkins

**Affiliations:** ^1^ Epworth Radiation Oncology Epworth Hospital Richmond Victoria Australia; ^2^ Research Development and Governance Epworth Healthcare Melbourne Australia; ^3^ School of Public Health and Preventive Medicine Monash University Melbourne Australia

**Keywords:** calypso, cone‐beam computed tomography, electromagnetic transponders, localization, prostatic neoplasms, radiographic imaging, radiotherapy

## Abstract

The aim of this study was to evaluate the differences in target localization between Calypso^®^, kV orthogonal imaging and cone‐beam computed tomography (CBCT) for combined translations and rotations of an anthropomorphic pelvic phantom. The phantom was localized using all three systems in 50 different positions, with applied translational and rotational offsets randomly sampled from representative normal distributions of prostate motion. Lin's concordance correlation coefficient (*ρ*c) and 95% confidence intervals were calculated to assess the agreement between the localization systems. Mean differences and difference vectors between the three systems were also calculated. Agreement between systems for lateral, vertical, and longitudinal translations was excellent, with *ρ*c values of greater than 0.98 between all three systems in all axes. There was excellent agreement between the systems for rotations around the lateral axis (pitch) (*ρ*c > 0.99), and around the vertical axis (yaw) (*ρ*c > 0.97). However, somewhat poorer agreement for rotations around the longitudinal axis (roll) was observed, with the lowest correlation observed between Calypso and kV orthogonal imaging (*ρ*c = 0.895). Mean differences between the phantom position reported by Calypso and the radiographic systems were less than 1 mm and 1° for all translations and rotations. The results for translations are consistent with the publications of previous authors. There is no comparable published data for rotations. While there is lower correlation between the three systems for roll than for the other angles, the mean differences in reported rotations are not clinically significant.

## 1 INTRODUCTION

One of the greatest difficulties in the provision of external beam radiation therapy treatment is correcting for tumor and organ motion to ensure accurate dose delivery. For prostate radiotherapy, previous studies have shown that the shape and position of the target varies from day to day (interfraction motion) and during treatment (intrafraction motion), due to variability in patient setup, bladder and bowel filling, and patient respiration.^1^, ^2^ The prostate gland is liable to nontrivial combined intra‐ and interfractional movements and rotations, with most motion occurring in the anteroposterior and superoinferior planes, and around the left‐right axis (pitch).^1^, ^2^, ^3^, ^4^, ^5^

In current radiotherapy practice, various methods of target localization are used to correct for prostate motion.^6^ Standard practice in Australia is to use radiographic imaging to visualize radiopaque fiducial markers implanted into the prostate.^2^, ^7^ The most commonly used imaging modalities are orthogonal kilovoltage planar x‐rays (kV‐imaging) and kilovoltage cone‐beam computed tomography (CBCT). Images are usually taken prior to treatment delivery, and hence correct for interfraction motion but not intrafraction.

An alternative localization method is to use electromagnetic transponders implanted in the prostate, which can be monitored via low frequency radio waves instead of ionizing radiation. Systems using electromagnetic transponders, such as the Calypso^®^ 4D Localization System, are being increasingly used to correct for both inter‐ and intrafraction prostate motion using target localization and real‐time tracking of implanted transponders.^8^, ^9^

Balter et al.^10^ evaluated the accuracy and precision of this electromagnetic transponder system for translational offsets using a precisely machined mechanical jig to control the transponder position. They found that both accuracy and precision decreased with increasing distance between the transponders and the detector array, but both were less than 1 mm in all three axes over the range of geometries tested.

Other authors have compared transponder‐based systems with radiographic imaging in phantom studies.^11^, ^12^, ^13^, ^14^ Santanam et al. (2009)^12^ compared Calypso with kV orthogonal imaging in a phantom, finding agreement within 1 mm in all axes. Ogunleye et al.^13^ also compared Calypso with kV orthogonal imaging in a phantom study, and reported submillimeter agreement between the two systems.

Patient and animal studies have also demonstrated strong correlation between transponder‐based systems and radiographic systems.^5^, ^11^, ^13^, ^15^, ^16^, ^17^, ^18^ Foster et al.^15^ compared Calypso with CBCT and kV orthogonal imaging over 900 and 250 fractions respectively, and found the mean differences in localization were less than 1 mm in all axes. Ogunleye et al.^13^ extended their phantom study to a cohort of 259 patient measurements, again demonstrating good correlation between kV orthogonal imaging and Calypso. The results of Willoughby et al.^16^ were similar, with a mean 3D distance vector difference of 1.5 mm between Calypso and orthogonal kV‐imaging. Quigley et al.^17^ compared Calypso with radiographic monitoring over 1027 fractions using the ExacTrac system and found that the mean vector length difference between Calypso and Exactrac was 1.9 mm ± 1.2 mm. Commissioning tests performed in our own center showed similar agreement between Calypso and radiographic imaging for translations, with mean 3D distance vector differences of 1.1 mm and 1.5 mm for Calypso‐kV orthogonal and Calypso‐CBCT, respectively.

While the studies described above have demonstrated strong correlation between transponder‐based systems and radiographic systems for translational offsets, little has been published about the accuracy of rotational offsets. Santanam et al. (2009)^12^ investigated the accuracy of Calypso rotation as part of their commissioning procedure. They tilted a phantom through 20° of pitch and roll by positioning it on a foam wedge, and compared the rotational values reported by Calypso with those recorded using a digital level. They tested yaw by rotating the treatment couch. The authors’ reported agreement within 1° for all rotations, but only tested five positions in total (two each for pitch and roll, one for yaw). Commissioning tests conducted in our own center using similar methods showed similar results. Li et al. (2009)^19^ also checked the rotational accuracy of Calypso in a phantom and reported that it was within 1°, but did not provide details of their methods or results.

In spite of this paucity of evidence evaluating the accuracy of transponder‐based systems like Calypso in measuring rotational offsets, the authors note that a number of recent studies have used Calypso‐generated localization data to draw conclusions on margin calculations and dosimetric coverage.^20^, ^21^, ^22^, ^23^ For example, the results of one study using this rotational offset information suggested that inter‐ and intrafraction prostatic rotations may result in target underdosing in up to 61% of patients depending on the PTV expansion used.^20^ The same study above also proposed that this information may be useful in the future in developing a metric to predict target coverage in the clinic prior to radiotherapy treatment.

In consideration of the current and future usage of Calypso‐derived rotational offset information, the aim of this phantom study was therefore to determine the level of agreement between Calypso with both kV orthogonal imaging and cone‐beam CT for a series of realistic combined rotations and translations, representative of typical prostate motion as described in the literature.^3^, ^24^

## 2 METHODS AND MATERIALS

### 2.A Study design

This was an observational quality assurance (QA) study to assess the accuracy of the Calypso^®^ 4D localization system compared with CBCT and kV orthogonal imaging. Numerous statistical analyses are available to determine the level of agreement between two methods of measuring the same continuous variable. Conventional measures of correlation, such as Pearson's, can achieve their maximum value of +1/‐1 even when there is no agreement between measures (i.e., the Pearson or Spearman correlation between scores 1,2,3 and 4,5,6 is 1, yet no pair of scores is equal). Other measures, such as the intraclass correlation, and the more recent Lin's concordance coefficient (*ρ*c),^25^, ^26^ only achieve a value of 1 when two sets of scores are identical to each other.

In line with McBride's^27^ recommendations, Lin's concordance correlation was used to evaluate agreement between the localization systems for this study. As such, based on the results of a previous study,^15^ a concordance correlation coefficient of at least 0.80 between each pair of techniques was expected. As sample size calculators based directly upon the expected confidence interval for a concordance correlation coefficient are not widely available, sample sizes were based upon the broadly similar intraclass correlation coefficient, with an expected value of 0.80, and 80% assurance of obtaining a 95% confidence.^28^

Previously published studies of internal prostate movement confirmed that rotations and translations follow approximately normal distributions, and provided values for the associated means and standard deviations.^3^, ^24^ Three sets each of 50 translational and 50 rotational offsets were randomly sampled from normal distributions generated using the aforementioned specified means and standard deviations from references^3^, ^24^ using Monte Carlo methods, obtained using the RANNOR function of SAS 9.4 (SAS Institute Incorporated, Cary, North Carolina, 2013). These 50 sets of values were used to generate the table of phantom translations and rotations used for the study (Table S1).

### 2.B Simulation and planning

**Figure 1:**
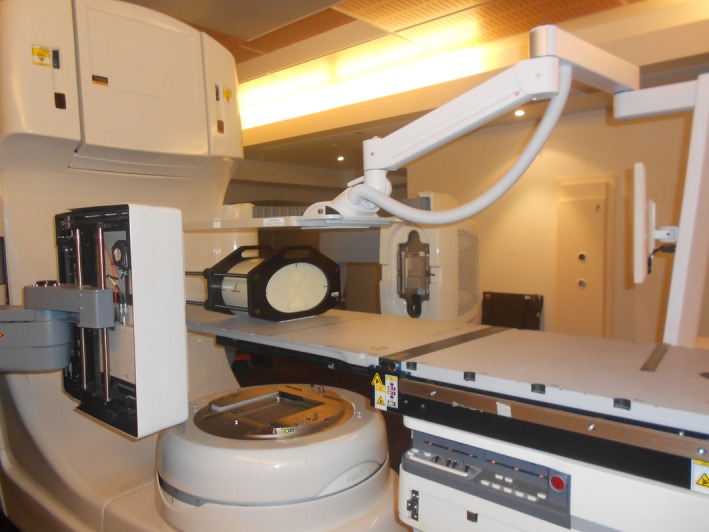
CIRS pelvic phantom.

The Calypso system has previously been described in detail.^10^, ^17^ For this study, three Calypso beacon transponders were inserted into a radiolucent foam cylinder representing the prostate gland. The transponders were positioned in the shape of an equilateral triangle with side length approximately 3 cm, in accordance with the manufacturer's recommendations. The foam cylinder was subsequently placed inside an anthropomorphic pelvic phantom (CIRS pelvic phantom) (Fig. 1). The phantom was scanned on a Siemens SOMATOM Emotion CT scanner (Siemens Medical Systems, Forchheim, Germany) with 1 mm slices.

Images were transferred to Varian's Eclipse™ treatment planning system (Version 11.0.42) (Varian Medical Systems, Palo Alto, California). Following standard planning procedures for Calypso patients, a treatment plan was created with the isocenter at the center of mass of the transponders. CBCT and orthogonal kV setup fields were added to the plan, to allow radiographic acquisition at the linear accelerator. Standard departmental procedures were used to transfer the treatment plan and associated imaging fields to Varian's Aria record and verify system (Version 13.6) and the Calypso tracking station (Version 3.0).

### 2.C Phantom positioning, localization, and image acquisition

The pelvic phantom was positioned and imaged 50 times for this study according to the set of pregenerated rotational and translational offsets (Table S1). Prior to localization, the phantom was placed on a board on the treatment couch top. The desired yaw was achieved by rotating the phantom on the board about the collimator rotation axis, using an angular scale marked on the phantom with the sagittal laser as a reference. The desired pitch and roll were achieved by tilting the board by placing spacers underneath it. Pitch and roll were measured using a 2‐axis digital level (Digi‐Pas DWL3000xy, Digipas Technologies Inc., Irvine, California). The position of the phantom on the board and the tilt of the board were iteratively adjusted until pitch, yaw, and roll were each within 0.5° of the values specified in the table.

After applying the desired angular offsets, the phantom was positioned at the isocenter according to Calypso, as is normal clinical practice. While any localization system could have been used for this initial positioning, Calypso was used because it was the quickest method. The desired translational offsets were then applied by moving the treatment couch in the lateral, vertical, and longitudinal directions. The setup procedure resulted in the entire phantom being rotated and translated, rather than an internal rotation and translation of the prostate within the phantom. This was done mainly for measurement efficiency, since moving the prostate internally would have required dismantling and re‐assembling the phantom for each measurement. Moreover, it would not be possible to set internal yaw and pitch rotations since the foam “prostate” must fit within a machined cylindrical cavity inside the phantom.

Once the phantom was in the required location, the transponders were localized according to Calypso, with offsets recorded from the console screen. These values were subsequently verified by the associated session reports. Radiographic images were then acquired using the On‐Board Imager (OBI) (Version 1.6) and saved for later analysis. The images consisted of an orthogonal kV pair (right lateral and PA), and a full‐fan CBCT using the “Pelvis spotlight” protocol. This protocol uses a small field of view which provides high resolution in the central pelvis at the cost of missing some of the skin surface. All data were acquired on the same linear accelerator, a Varian Trilogy™. The 50 measurements required for the study were performed on three separate days over a twelve‐week period, with four measurements performed on the first day, 32 on the second and 14 on the last day.

### 2.D Radiographic image analysis

**Figure 2:**
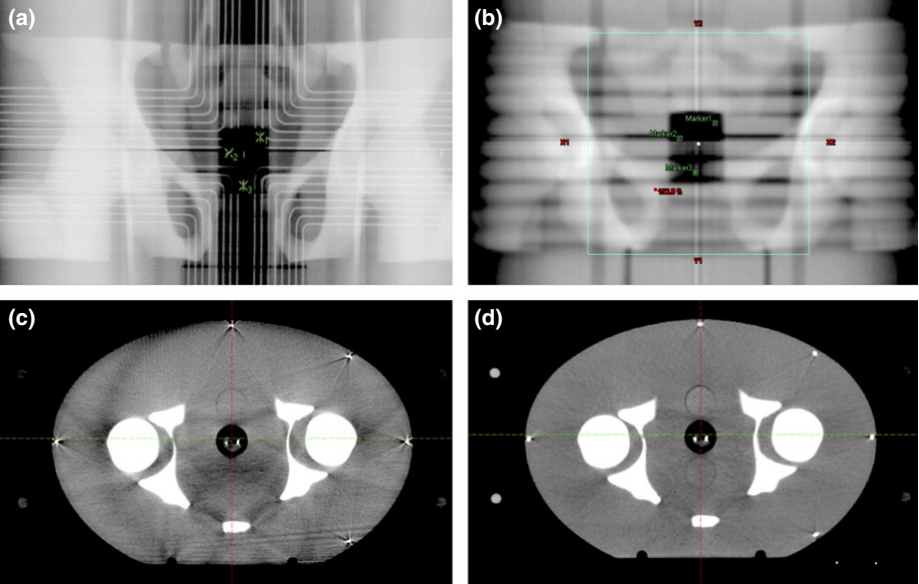
(a) Anterior kV image showing the Calypso transponders (green crosses) and copper wires in electromagnetic array, (b) planning DRR with markers indicating Calypso transponders, (c) CBCT, and (d) planning CT scan.

The kV orthogonal images and CBCT images were analyzed offline using the Varian™ Offline Review software (Version 13.6) (Fig. 2). For the purposes of the study, the software was configured to report rotational offsets about all three axes (pitch, roll, and yaw), although in our normal clinical practice only yaw is reported.

For the kV orthogonal images, the Marker Match option was used (Fig. 2). This matching algorithm allows the user to mark the position of fiducial markers in a pair of orthogonal images. The software then calculates translational and rotational offsets in three dimensions for the image pair. The centers of the three Calypso transponders were used as the fiducial markers. For the CBCT, the automated anatomy matching software was used. All the anatomy including bone and soft tissue analogs was used. The field of view for matching was set to encompass just the CBCT image, approximately 2 cm inside the phantom surface. The automated anatomy matching algorithm uses a mutual information algorithm to align the CBCT with the planning CT data set, again reporting translational and rotational offsets in three dimensions. Both of these matching algorithms were chosen specifically in order to minimize the operator‐dependent uncertainty in matching, which would be greater if manual matching was used.

All images were acquired, matched and entered by one author (AEP) into a preprepared spreadsheet, then checked for accuracy by the primary author (DGH). As Calypso and Aria use different conventions for reporting rotations and translations, all translation and rotation values from the radiographic imaging systems were corrected to match the Calypso conventions, which are as described in Santanam et al. (2009).^12^

### 2.E Statistical analysis

Lin's concordance correlation coefficients (*ρ*c) and 95% confidence intervals were calculated to assess the degree of correlation between Calypso‐kV, Calypso‐CBCT, and kV‐CBCT localizations. The differences between Calypso‐kV, Calypso‐CBCT, and kV‐CBCT were calculated for all three translational axes (lateral, vertical, and longitudinal) and all three rotations (pitch, roll, and yaw). The mean difference, standard deviation, and range for the 50 measurements were then derived. The vector length differences between the three imaging systems were also calculated.^13^ Offset values were generated using SAS (Version 9.4, SAS Institute Incorporated, Cary, North Carolina, 2013) as described above. All other analyses were performed using Stata (version 14) (Stata Corporation, College Station, Texas, 2015).

As described above, Lin's concordance correlation coefficient is used to determine the level of agreement between two methods of measuring the same continuous variable. Different authors have made suggestions for interpreting the scores for Lin's concordance correlation coefficients.^27^, ^29^ One such proposal by McBride,^27^ in assessing the degree of equivalence between new laboratory tests and the gold standard, has suggested that a lower 95% confidence interval greater than 0.99 represents almost perfect agreement, 0.95 to 0.99 substantial agreement and 0.90 to 0.95 moderate agreement. Although McBride^27^ was considering a different specific application (laboratory tests in microbiology), his criteria appear to be appropriate for this study.

### 2.F Quality assurance checks of localization systems

During the period of the study, the coincidence between the lasers, Calypso, the kV‐imaging system and the MV isocenter was checked according to standard departmental monthly quality assurance procedures. These are based on the recommendations of AAPM Task Group 142^30^ for a linear accelerator used for IMRT and SRS/SBRT. The Task Group 142 report does not give recommendations for QA of electromagnetic transponder systems. Because Calypso is an alternative localization system, we apply the same tests and tolerances to Calypso as to the kV‐imaging systems. Briefly, the QA procedure consists of locating the treatment radiation isocenter via a Winston‐Lutz test, and then confirming that all the localization systems (lasers, kV planar imaging, CBCT and Calypso) indicate the radiation isocenter within 1 mm in any axis. All QA tests were within tolerance and so no adjustments were made to any of the localization systems during the course of the study.

## 3 RESULTS

### 3.A Translations

**Figure 3:**
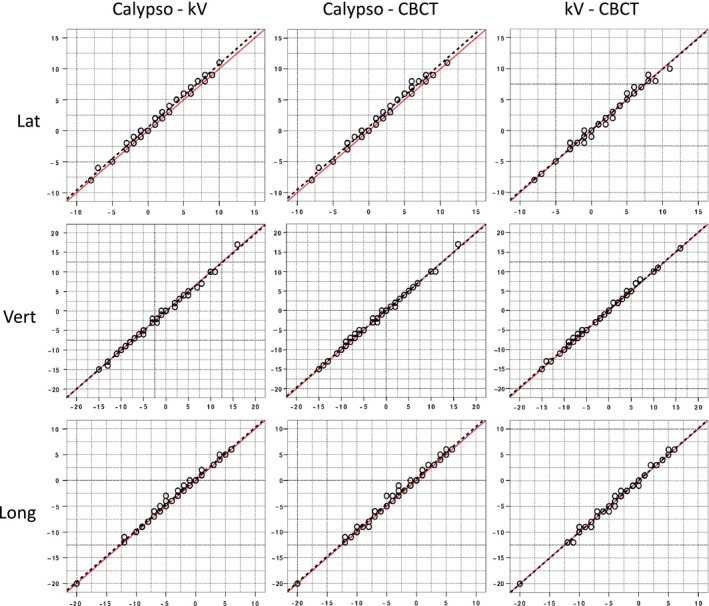
Scatter plots comparing all three localization systems for 50 measured lateral (top), vertical (middle), and longitudinal (bottom) translations. (Red lines refer to the line of identity and dashed black lines to the corresponding linear regression line.)

Scatter plots showing the translational offsets reported by Calypso and the radiographic imaging systems are shown in Fig. 3. Also shown in Fig. 3 are corresponding linear regression lines and the line of identity. The line of identity is the line along which all data would fall if there was perfect agreement between the systems. The linear regression line and line of identity are virtually indistinguishable in all the plots except for the lateral axis where there is an offset of approximately 0.6 mm between Calypso and the radiographic systems. Error bars are not shown on the graphs for reasons of clarity. Please refer to the appendix for a discussion of the uncertainties associated with the data in this figure.

Lin's concordance correlation coefficients (*ρ*c) between the three systems for translational localizations, with the associated 95% confidence intervals, are displayed in the upper half of Table 1. Agreement between all three systems in the vertical and longitudinal directions is excellent, with observed *ρ*c values of greater than 0.990 for all comparisons. In the lateral direction, there is also excellent correlation between kV orthogonal and CBCT (*ρ*c = 0.992, 95% CI 0.986–0.995). However, there is lower agreement between Calypso‐kV orthogonal (*ρ*c = 0.983, 95% CI 0.972–0.990) and Calypso‐CBCT (*ρ*c = 0.981, 95% CI 0.969–0.988).

**Table 1:** Lin's concordance correlation coefficient (*ρ*c) and 95% confidence intervals for localization values between Calypso‐kV imaging, Calypso‐CBCT and kV imaging‐CBCT for selected translations (mm) and rotations (deg)

	Calypso‐kV imaging		Calypso‐CBCT		kV imaging‐CBCT	
	*ρ*c	95% CI	*ρ*c	95% CI	*ρ*c	95% CI
Lateral	0.983	0.972–0.990	0.981	0.969–0.988	0.992	0.986–0.995
Vertical	0.997	0.995–0.999	0.998	0.996–0.999	0.998	0.996–0.999
Longitudinal	0.994	0.990–0.997	0.992	0.986–0.995	0.996	0.993–0.998
Pitch	0.995	0.992–0.997	0.995	0.991–0.997	0.994	0.989–0.996
Roll	0.895	0.830–0.936	0.947	0.914–0.967	0.939	0.896–0.965
Yaw	0.975	0.957–0.985	0.989	0.980–0.993	0.968	0.946–0.981

CBCT, cone‐beam computed tomography; kV, kilovoltage; CI, confidence interval.

Differences in translations between the three imaging systems are summarized in the upper half of Table 2, which shows the mean, standard deviation, and range for translational differences in the lateral, vertical, and longitudinal axes over the 50 measurements. The mean, standard deviation, and range for the difference vector length are also shown. The mean differences are all less than 1 mm in all three axes, as are the upper and lower 95% confidence intervals (results not reported). The maximum difference observed in any axis was 2 mm. The mean difference vector length is 1 mm or less in all cases, and the maximum length is 2.5 mm.

**Table 2:** Mean differences in localization between Calypso‐kV imaging, Calypso‐CBCT and kV imaging‐CBCT for random translations (mm) and rotations (deg)

	Calypso‐kV imaging			Calypso‐CBCT			kV imaging‐CBCT		
	Mean	SD	Range	Mean	SD	Range	Mean	SD	Range
Lateral	0.6	0.5	(0, 1)	0.6	0.5	(0, 2)	0.0	0.5	(−1, 1)
Vertical	−0.1	0.5	(−1, 1)	0.1	0.5	(−1, 1)	0.2	0.4	(0, 1)
Longitudinal	0.3	0.5	(0, 2)	0.3	0.6	(−1, 2)	0.0	0.5	(−1, 1)
ΔVector	0.9	0.5	(0, 2.2)	1.0	0.6	(0, 2.5)	0.6	0.6	(0, 1.4)
Pitch	0.0	0.4	(−1, 1)	−0.1	0.4	(−1, 1)	−0.1	0.5	(−1,1)
Roll	0.4	1.0	(−1, 3)	0.1	0.7	(−2, 2)	−0.3	0.8	(−2,1)
Yaw	−0.2	0.5	(−1, 1)	0.1	0.3	(0, 1)	0.3	0.5	(−1,1)

CBCT, cone‐beam computed tomography; kV, kilovoltage; SD, standard deviation; ΔVector, difference vector magnitude.

### 3.B Rotations

**Figure 4:**
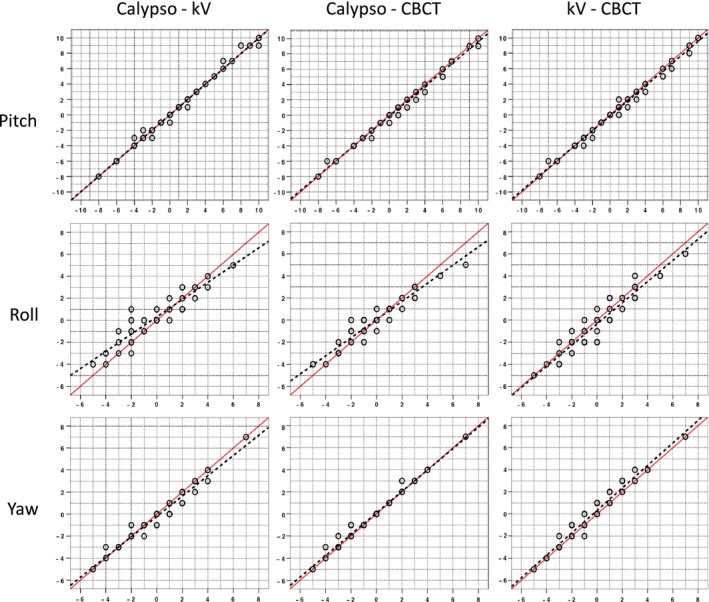
Scatter plots comparing all three localization systems for 50 measured pitch (top), roll (middle), and yaw (bottom) rotations. (Red lines refer to the line of identity and dashed black lines the corresponding linear regression line.)

Scatter plots showing the rotational offsets reported by Calypso and the radiographic imaging systems are shown in Fig. 4, with the linear regression line and the line of identity. The two lines are virtually indistinguishable for pitch, but for roll there is a noticeable difference in slope, particularly when comparing Calypso with the radiographic imaging systems. There is also a difference in slope of the two lines for yaw when comparing Calypso with kV orthogonal. Error bars are not shown in Fig. 4 for clarity, but sources of uncertainty are discussed in the appendix.

Concordance correlation coefficients (*ρ*c) between all three systems for rotational localizations are displayed in the lower half of Table 1. Agreement between all three systems for pitch was excellent, with *ρ*c values greater than 0.990 in all cases. For yaw, the degree of correlation is less, with *ρ*c values in the range 0.968–0.989. The degree of correlation with roll is noticeably poorer, with *ρ*c values of 0.895 for Calypso‐kV imaging, 0.947 for Calypso‐CBCT, and 0.939 for kV imaging‐CBCT.

Differences in rotations between the three imaging systems are summarized in the lower half of Table 2, which shows the mean, standard deviation, and range for differences in pitch, roll, and yaw. The mean differences are less than 1° in all three cases. The maximum difference observed for any rotation was 3°, which was recorded when comparing Calypso with kV orthogonal imaging for roll.

## 4 DISCUSSION

The aim of this phantom study was to prospectively compare the accuracy of Calypso with kV orthogonal imaging and CBCT in detecting combined translations and rotations over a range of offsets representative of typical prostate motion. Unlike previously published studies, the current work has investigated rotational accuracy in detail, and in combination with translations.

### 4.A Translations

Our results for translations are similar to those published by other authors in phantom and patient studies.^13^, ^15^, ^16^, ^18^ Table 3 shows our mean differences and standard deviations for translations in the lateral, vertical and longitudinal directions, and for the vector translation, in comparison with the results of these studies. The results of all studies are in agreement within two standard deviations. The highest mean difference in any axis reported by any study is 1.2 mm,^13^ and all other studies show submillimeter agreement.

**Table 3:** Previously reported mean differences in localization (magnitude only) between Calypso and radiographic imaging for translations (mm)

Author	Study type	N	Radiographic comparator	Mean difference (SD)			
				Lat	Vert	Long	Vector
Current study	Phantom	50	kV‐imaging	0.6 (0.5)	0.1 (0.5)	0.3 (0.5)	1.0 (0.5)
	Phantom	50	kV‐CBCT	0.6 (0.5)	0.1 (0.5)	0.3 (0.6)	1.0 (0.6)
Willoughby^16^	Phantom	NR	kV‐imaging	–	–	–	0.5 (0.1)
	Patient	11	kV‐imaging	–	–	–	1.5 (0.9)
Ogunleye 13	Phantom	30	kV‐imaging	0.4 (0.4)	0.2 (0.3)	0.4 (0.3)	0.8 (0.4)
	Patient	259	kV‐imaging	0.7 (0.5)	1.2 (0.9)	1.1 (0.9)	2.1 (1.0)
Kupelian 18	Patient	1027	ExacTrac	0.1 (0.9)	0.0 (1.3)	0.4 (1.4)	1.9 (1.2)
Foster 15	Patient	260	kV‐imaging	0.1 (1.0)	0.1 (1.4)	0.5 (1.5)	–
	Patient	915	kV‐CBCT	0.0 (1.2)	0.2 (2.9)	0.8 (2.2)	–

SD, standard deviation; kV, kilovoltage; CBCT, cone‐beam computed tomography.

Although there is good agreement between all the results in Table 3, the data suggest that some institutions achieve better mean agreement between Calypso and their radiographic imaging systems than others, and that agreement is better in some directions than others. This is supported by our own data, which shows evidence of a small offset in the lateral direction between Calypso and the radiographic imaging systems (see Fig. 3). We believe that this represents a small systematic error of approximately 0.6 mm in the calibration of our localization systems, although well within our monthly QA tolerance of 1 mm.

While these results are encouraging, the data presented in this report only enable us to identify a discrepancy between Calypso and the radiographic systems — it does not tell us how the systems compare to the megavoltage radiation isocenter, which is the fundamental reference for calibration of localization systems. This could be determined by performing the monthly quality assurance check described in the Methods section. When multiple localization systems are available in a department, there may be a need to consider tighter tolerances on each individual system than specified in the AAPM TG142 report.^30^ In the extreme case, two localization systems may each be within 1 mm of the megavoltage radiation isocenter and hence within the AAPM TG142 tolerance, but may differ by 2 mm from each other. Such disagreement can pose clinical decision‐making problems when systems are used together for patient localization, particularly when tight treatment margins are used.

Table 3 also shows that the standard deviations are generally lower in the phantom studies than in patient studies. This is not surprising. In the patient studies, intrafraction motion secondary to respiration and bladder and bowel filling will contribute to differences in transponder position if the radiographic imaging and Calypso monitoring are performed at different times. Furthermore, the algorithms used by Calypso and the radiographic systems to calculate patient position may handle rotations and distortions differently, contributing to increased uncertainty in the reported positions.

### 4.B Rotations

Our data on rotations are, so far as we are aware, the first published which systematically compares the rotational accuracy of Calypso with radiographic imaging over a range of angular offsets. We have found overall excellent agreement between Calypso and the radiographic systems for pitch, roll, and yaw over a range of clinically relevant angles. The mean differences between each system are less than 1°, and the largest discrepancy was 3°. These data are consistent with the results reported by Santanam et al. (2009)^12^ and Li et al. (2009).^19^ However, the Lin's concordance correlation coefficient results show that agreement between the systems is best for pitch, somewhat worse for yaw, and worse again for roll. The biggest disagreement was found between Calypso and kV orthogonal imaging for determination of roll (*ρ*c = 0.895, see Table 1). It is difficult to be certain of the reasons for this without full knowledge of the algorithms used by all the systems to determine rotations. We believe that one factor is a fundamental limitation of matching using orthogonal kV images. Roll is a rotation around the longitudinal axis, which is also the axis around which the gantry rotates when taking orthogonal kV images. Visual inspection of kV orthogonal images shows that yaw (rotation about the vertical axis) is readily apparent in a PA image, and pitch (rotation about the lateral axis) is readily apparent in a lateral image, but roll is only indirectly visible as changes in the apparent separation of the fiducial markers in both the AP and lateral images. Even without knowing the details of the marker match algorithm, it is not surprising that roll may be partially misinterpreted as translation when using orthogonal image pairs. This suspicion is given additional weight by our uncertainty analysis, which shows a greater uncertainty in the kV orthogonal matching for roll than for pitch and yaw (see appendix).

Detection of roll in CBCT images should not be affected in the same way as kV orthogonal pairs, since the matching algorithm uses the full 3D data set. This is also confirmed by our uncertainty analysis (see appendix). Further confirmation comes from inspection of our raw experimental data (not presented here) which shows that CBCT gave results that matched our digital level readings within 1° for all roll settings, whereas both Calypso and kV orthogonal matching differed from the digital level by up to 2°.

### 4.C Lin's concordance correlation coefficient

Applying the criteria described by McBride^27^ to our results indicates that we have “almost perfect agreement” between the three localization systems for vertical and longitudinal translations. This is in agreement with the data shown in Fig. 3 and Table 2, which confirms that the systems give essentially equivalent results. McBride's^27^ terminology of “almost perfect agreement” could be interpreted in the radiotherapy context as meaning that a patient could be positioned with any of the systems with the same level of accuracy.

For lateral translations, our data comparing Calypso to the radiographic systems would fall into the “substantial agreement” category. As noted previously, there appears to be a small offset of approximately 0.6 mm between Calypso and the radiographic imaging systems (see Table 2 and Fig. 3). Although the systems have an equivalent ability to detect translational offsets, there is likely, however, a systematic difference between them attributable to a different reference point being established at the time the systems were calibrated. In the context of this study, “substantial agreement” could be interpreted as “within calibration tolerances”. Applying these criteria to our rotation data indicates almost perfect agreement for pitch, substantial agreement for yaw but only moderate agreement for roll.

### 4.D Study limitations

While this study was successful in its aim of systematically comparing Calypso with radiographic imaging for a range of clinically relevant combined rotations and translations, it does have a number of limitations.

Because the aim was to investigate the capabilities of all the systems under the best possible circumstances, we used a number of procedures that are not part of our normal clinical practice for prostate patients including:

1. Using the Marker Match algorithm for the kV orthogonal images, when manual matching is our normal clinical practice.
2. Using automatic anatomy matching for the CBCT images, when manual matching to markers is our normal clinical practice.
3. Reporting roll, pitch and yaw for the radiographic images (both kV orthogonal and CBCT), when in clinical practice only yaw is reported.

The results of our study would be expected to differ if we used our normal clinical match settings. Results may also differ if a different individual performed the matching. Further investigations are planned to test the impact of these practices.

Although we used a pelvic phantom for the study, in order to achieve a realistic patient geometry for testing the systems, our experimental setup had some limitations. One of these was that we chose to rotate and translate the whole phantom rather than moving the prostate internally. As noted in the methods section, this was done primarily for practical reasons, to enable the measurements to be carried out in a reasonable time frame without the need to disassemble the phantom for each measurement. Although rotating the whole phantom is not representative of normal patient treatment, we feel that this is was not of major significance for our phantom based study, since in a phantom‐based study the relationship of all components of the phantom remains fixed.

Another potential phantom‐related limitation of this study is the relatively small size of the CIRS pelvic phantom (20 cm in the anteroposterior direction by 30 cm in the lateral direction). Calypso cannot be used for men with a substantially large body habitus, as the manufacturer has built in a software restriction that limits the maximum distance between the detector array and the transponders. Investigations performed during commissioning of Calypso in our center showed that phantom size did not significantly affect the positional accuracy of Calypso over the range of allowed array distances, although random noise in the Calypso signal increased slightly for larger phantoms, as the distance between the transponders and the detector panel increased. These results are in agreement with those reported by Balter et al.^10^ who found submillimeter accuracy was maintained as distance from the array to the transponders was increased up to the maximum allowed value of 27 cm. In theory, the accuracy of radiographic imaging would be expected to decrease slightly with phantom size due to reduced signal to noise ratio, but our clinical experience shows that this is not significant for patients eligible for Calypso treatment.

Another limitation of our experimental setup is that the Calypso beacons and isocenter were positioned as per manufacturer guidelines. In patients, such ideal placement of the beacons and isocenter is not always possible, and under such situations we would expect the level of agreement between the localization systems to worsen as the beacon geometry becomes more collinear and the separation of the beacons reduces. Positioning the isocenter away from the beacon center of mass would not affect our results however. All three systems can still detect the beacon position regardless of the isocenter location, provided that the beacons do not move outside the system's field of view.

Furthermore, we kept the beacons fixed in the same spatial relationship to each other throughout the experiment. Literature shows that in patients, fiducial markers implanted in the prostate can migrate, as well as move relative to each other.^2^, ^31^ Fiducial deformation is also commonplace among patients receiving postprostatectomy radiotherapy where transponders are inserted into the prostatic fossa.^32^ No attempt was made in this study to assess the effects of deformations and transponder movement. It is expected that the algorithms used by Calypso and the radiographic imaging will handle deformations differently, and hence there are likely to be greater discrepancies between the systems in patients than in this idealized phantom geometry, as reflected in the publications of other authors summarized in Table 3.

### 4.E Clinical implications

The results of our study confirm the findings of others that Calypso gives a similar level of accuracy for patient translations as current best practice radiographic imaging.^13^, ^15^, ^16^ In addition, we now have increased confidence in the accuracy of the rotational information provided by Calypso. This information is important for our center in establishing the reliability of Calypso, so that our patients can benefit from the real‐time intrafraction motion detection. It also opens up the possibility of decreasing the use of radiographic imaging, thereby reducing patient imaging dose with negligible impact on treatment efficiency.^17^

In theory, when a rotation is detected, this could be corrected for by changing patient tilt position (e.g., using a 6D couch). This however is not standard practice for prostate radiotherapy, although it is commonly used for stereotactic work. The negative impact of prostate rotation on dosimetric coverage has been widely recognized.^20^, ^21^, ^33^ Consequently, the discovery of large rotational offsets upon prostate localization in the clinic will impact clinical decision‐making. When Calypso detects rotation outside the predefined limits, it warns the operator. In our center, this warning serves as a trigger to investigate patient set up and to perform soft tissue imaging to assess bladder and bowel filling.

To date, there have been several studies that have utilized Calypso‐generated rotational offsets to evaluate PTV margins and dosimetric coverage for prostate radiotherapy.^20^, ^21^, ^22^, ^23^ These studies show that target rotations can cause significant target under‐dosing even when translations are corrected for, with negative effects complexly linked to a combination of the magnitude of rotation, target shape and the distance between the target and transponders’ rotational centroid (eccentricity).^20^, ^22^ As such, while there is not yet a universally recognized trigger value for action, validation of the integrity of Calypso‐derived rotational offset information is valuable, particularly if these values are to be used in the future as evidence to assist in clinical decision‐making.

## 5 CONCLUSION

This phantom study has compared Calypso with kV orthogonal imaging and CBCT in measuring combined translations and rotations over a range of movements representative of typical prostate motion. Unlike previously published studies, the current work has investigated rotational accuracy in detail, and in combination with translations. Our results confirm the results of other authors with regard to translations, with submillimeter differences and substantial to almost perfect agreement observed between the three systems. For rotations, varying degrees of agreement were observed depending on the rotational axis. In spite of this, mean differences were less than 1° for all three systems, which is adequate for clinical use. These results give confidence in the use of Calypso translational and rotational data for patient positioning, margin calculation, and treatment decision‐making.

## ACKNOWLEDGMENTS

The authors acknowledge and thank the Australian Clinical Dosimetry Service for provision of the CIRS phantom.

## CONFLICTS OF INTEREST

The authors report no conflicts of interest or financial interests. The authors alone are responsible for the content and writing of the paper.

## RESEARCH FUNDING

This work was supported by the Epworth Research Institute [grant number 11.952.000.80992].

## Supporting information


**Table S1.** List of randomly generated translational (mm) and rotational offsets (degrees).
**Table S2.** Combined uncertainty in reported translations (mm) and rotations (degrees) for Calypso and radiographic imaging. Uncertainties calculated according to the ISO GUM at the 95% confidence level.

## APPENDIX 1

### UNCERTAINTY IN MATCHING

The main contributor to the uncertainty in reported translations and rotations was rounding errors. The radiographic matching software only reports translations to the nearest millimeter, and although Calypso reports positions to one tenth of a millimeter, the Calypso results were rounded to the nearest millimeter prior to statistical analysis. Calypso only reports angles to the nearest whole degree. The radiographic matching software reports angles to tenths of a degree, but the values were rounded to the nearest degree prior to analysis.

For radiographic imaging, a second contributor to uncertainty is operator‐dependent variation in matching. For kV orthogonal imaging, this was assessed by performing 20 independent matches on the same image set and calculating the standard deviation of reported translations and rotations. For translations, all 20 matches gave identical values (within the 1 mm resolution of the software), resulting in a standard deviation of 0 mm. For rotations, the standard deviations were 0.4°, 0.7°, and 0.4° for pitch, roll, and yaw, respectively.

A similar set of 20 independent matches was performed on a single CBCT data set. As for the kV orthogonal images, there was a standard deviation of 0 mm for translations. For rotations, the standard deviations were less than 0.1° for all three angles.

Calypso rotations and translations are not subject to operator‐dependent variation. However, there is random noise in the Calypso trace that contributes to uncertainty in the reported translations. This was assessed by assuming that the noise is normally distributed and estimating the standard deviation from the maximum excursion reported by the software. The estimated standard deviation was less than 0.1 mm in all three axes. Random noise is assumed not to contribute to uncertainty in reported rotations.

The combined uncertainties in reported translations and rotations were calculated according to the ISO GUM^34^ and are summarized in Table S2.
