# Supplementary material for: Comparison between electromagnetic transponders and radiographic imaging for prostate localization: A pelvic phantom study with rotations and translations
Source: J Appl Clin Med Phys. 2017 Jul 12;18(5):43–53. doi: 10.1002/acm2.12119 (PMC5875817; doi:10.1002/acm2.12119)
Supplement: Supplementary file 1 — Table S1. List of randomly generated translational (mm) and rotational offsets (degrees). Table S2. Combined uncertainty in reported translations (mm) and rotations (degrees) for Calypso and radiographic imaging. Uncertainties calculated according to the ISO GUM at the 95% confidence level. [file ACM2-18-43-s001.doc]

**SUPPLEMENTARY TABLES**

**Table S1.** List of randomly drawn translational (mm) and rotational offsets (degrees).

| **Measurement No.** | **Lateral** | **Vertical** | **Longitudinal** | **Yaw** | **Roll** | **Pitch** |
| --- | --- | --- | --- | --- | --- | --- |
| 1 | 7 | 9 | 9 | 4 | 1 | -10 |
| 2 | 9 | 2 | -1 | -3 | -3 | -6 |
| 3 | -1 | -17 | 3 | 1 | -5 | -1 |
| 4 | 6 | -3 | 7 | -1 | 3 | -3 |
| 5 | 0 | -2 | 1 | 0 | -1 | 3 |
| 6 | 0 | -2 | -1 | 0 | 1 | 0 |
| 7 | -8 | 9 | -3 | -1 | -3 | 4 |
| 8 | 8 | 16 | 5 | -1 | 6 | 1 |
| 9 | 2 | 7 | 9 | 0 | 2 | 2 |
| 10 | 5 | 9 | 2 | 1 | 0 | -6 |
| 11 | 1 | 10 | 0 | -6 | 0 | -1 |
| 12 | 0 | 0 | 8 | 1 | 2 | 0 |
| 13 | -2 | 5 | 5 | 1 | 2 | 1 |
| 14 | -5 | -10 | -5 | 0 | 0 | -1 |
| 15 | 4 | 3 | 1 | 0 | -2 | -1 |
| 16 | 0 | 8 | 8 | 1 | -3 | -4 |
| 17 | 7 | -10 | 10 | 0 | -3 | -3 |
| 18 | 5 | 1 | 3 | 2 | -1 | -4 |
| 19 | -2 | 5 | 0 | -3 | 2 | -9 |
| 20 | -1 | -10 | 9 | 2 | 1 | 1 |
| 21 | 6 | 13 | -4 | 0 | -2 | 3 |
| 22 | 2 | 9 | 6 | 1 | -1 | -1 |
| 23 | -1 | 5 | 10 | 2 | -2 | 2 |
| 24 | 0 | 6 | 9 | 1 | 4 | 2 |
| 25 | 6 | 0 | 0 | -1 | -4 | 0 |
| 26 | 0 | 10 | 0 | 4 | -3 | -1 |
| 27 | -2 | 0 | 2 | 2 | 2 | -7 |
| 28 | -1 | 6 | -4 | 2 | -3 | 1 |
| 29 | -1 | -4 | 3 | -2 | 1 | 2 |
| 30 | 7 | 3 | 4 | 1 | 2 | -1 |
| 31 | 9 | 0 | 4 | 3 | -2 | 8 |
| 32 | 3 | 4 | 3 | 2 | -1 | 7 |
| 33 | 12 | -6 | 4 | 1 | -2 | -2 |
| 34 | 1 | 7 | -4 | 0 | -2 | -7 |
| 35 | -1 | 8 | 11 | -1 | -1 | 3 |
| 36 | -1 | 8 | 6 | -3 | -1 | 6 |
| 37 | 4 | 11 | -6 | -1 | 0 | -1 |
| 38 | 9 | 2 | 1 | 3 | -1 | -10 |
| 39 | 2 | 13 | 19 | 0 | 0 | -1 |
| 40 | -3 | -2 | -1 | -2 | -1 | -2 |
| 41 | 3 | 3 | -2 | 0 | -3 | 2 |
| 42 | 5 | 0 | 6 | -1 | 2 | 4 |
| 43 | -5 | 0 | 1 | 3 | -1 | -1 |
| 44 | -2 | 2 | 1 | -2 | 0 | -1 |
| 45 | 5 | -1 | 10 | 1 | -2 | 6 |
| 46 | 2 | -4 | -6 | -1 | 1 | -1 |
| 47 | 2 | 8 | 11 | 1 | -1 | -2 |
| 48 | 1 | -1 | 5 | 1 | 1 | 4 |
| 49 | -1 | -5 | 1 | 2 | -1 | -9 |
| 50 | 8 | -7 | 6 | 0 | 0 | 3 |
| Mean (SD) (actual) | 2.1 (4.2) | 2.4 (6.8) | 3.3 (5.2) | 0.28 (1.91) | -0.44 (2.16) | -0.62 (4.24) |
| Mean (SD) (planned) | 0.9 (4.4) | 2.5 (5.8) | 2.7 (4.3) | 0.09 (2.01) | -0.52 (2.30) | -0.01 (3.95) |

**Table S2.** Combined uncertainty in reported translations (mm) and rotations (degrees) for Calypso and radiographic imaging. Uncertainties calculated according to the ISO GUM at the 95% confidence level.

kV orthogonal CBCT Calypso

ucomb DOF ucomb DOF ucomb DOF

Lateral 0.6 1000 0.6 1000 0.6 1236

Vertical 0.6 1000 0.6 1000 0.7 1915

Longitudinal 0.6 1000 0.6 1000 0.6 1408

Pitch 1.1 38 0.6 885 0.6 1000

Roll 1.5 26 0.6 1000 0.6 1000

Yaw 1.0 41 0.6 1000 0.6 1000

CBCT – cone-beam computed tomography, ucomb - combined uncertainty, DOF - degrees of freedom
